# New perspectives of robotic surgery are the topic highligheted in International Brazilian Journal of Urology

**DOI:** 10.1590/S1677-5538.IBJU.2023.06.01

**Published:** 2024-02-07

**Affiliations:** 1 Universidade do Estado do Rio de Janeiro Unidade de Pesquisa Urogenital Rio de Janeiro RJ Brasil Unidade de Pesquisa Urogenital - Universidade do Estado do Rio de Janeiro - Uerj, Rio de Janeiro, RJ, Brasil; 2 Hospital Federal da Lagoa Rio de Janeiro RJ Brasil Serviço de Urologia, Hospital Federal da Lagoa, Rio de Janeiro, RJ, Brasil

The Novermber-December number of *Int Braz J Urol* is the 25^nd^ under my supervision. In this number the Int Braz J Urol presents original contributions with a lot of interesting papers in different fields: Robotic Surgery, Prostate Cancer, Bladder Cancer, Ureteral stones,UPJ obstruction, Bladder dysfunction, Concealed Penis, Renal cell Carcinoma, Systematic review and Metanalysis. The papers came from many different countries such as Brazil, USA, Spain, Iran, Italy, Germany and China, and as usual the editors comment highlights some of them. The editor in chief would like to highlight some papers in this number, specially the papers about robotic surgery:

Dr. Belkovsky and collegues from Brazil, presented in page 668 (1) a nice systematic review about expulsive therapy for distal ureteral stones with Tamsulosin and Tadalafil and concluded that Tadalafil has a higher stone expulsion rate than tamsulosin as a medical expulsive therapy for patients with distal stones from 5 to 10 mm without differences in side effects.

Dr. Moschovas and collegues from USA, presented in page 677 (2) a important systematic review about the Outcomes of Salvage Robotic-assisted Radical Prostatectomy in the last decade and concluded that salvage robotic-assisted radical prostatectomy in patients with cancer recurrence after previous prostate cancer treatment is safe and feasible. The literature is based on retrospective studies with inherent limitations describing low rates of intraoperative complications and small blood loss. However, potency and continence rates are largely reduced compared to the primary RARP series, despite the type of the primary treatment. Better-designed studies to assess the long-term outcomes and individually specify each primary therapy impact on the salvage treatment are still needed. Future articles should be more specific and provide more details regarding the previous therapies and S-RARP surgical techniques.

Dr. Nunes and Collegues from Brazil performed in page 688 (3) a nice study about the Association between rectal diameter and response to treatment with parasacral transcutaneous electrical nerve stimulation and behavioral changes in children and adolescents with bladder and bowel dysfunction and concluded that rectal diameter appears to be relevant in the evaluation of children with BBD, not only as a diagnostic tool but also as a predictor of treatment outcome.

Dr. da Silva and Collegues form Brazil performed on page 700 (4) an interesting study about the Dynamic and static ultrasound (DSUS) features predictive of vesicoureteral reflux (VUR) and renal damage in children and adolecents with neurogenic bladder and concluded that DSUS predicts VUR and renal scarring in children with NB with fair to good accuracy, and all measurements exhibited a high negative predictive value (NPV). The increase in RPD during voiding or detrusor contractions proved to be the most accurate parameter for indicating the presence of VUR in this study.

Dr. Chen and Collegues form China performed on page 716 (5) a nice study about the Head-to-head comparisons of enhanced CT, 68Ga-PSMA-11 PET/CT and 18F-FDG PET/CT in identifying adverse pathology of clear-cell renal cell carcinoma and concluded that 68Ga-PSMA-11 PET/CT demonstrates distinct advantages in identifying aggressive pathological features of primary clear-cell renal cell carcinoma (ccRCC) when compared to enhanced CT and 18F-FDG PET/CT. Further research and assessment are warranted to fully establish the clinical utility of 68Ga-PSMA-11 PET/CT in ccRCC.

Dr. Bertolo and collegues from Italy performed on page 732 (6) a nice comparative analysis of perioperative outcomes about "Single-Surgeon" versus "Dual-Surgeon" in Robot-Assisted Radical Prostatectomy (RARP) and Pelvic Lymph-nodes Dissection (PLND) and concluded that when RARP and PLND are split onto two surgeons, the operative time is shorter by 20 minutes compared to when a single surgeon performs RARP and PLND.

Dr. Jing and collegues from China performed on page 740 (7) an study about a new treatment of concealed penis: symmetrical pterygoid flap surgery, this paper is the cover of the present edition. The authors concluded that the symmetrical pterygoid flap surgery is an effective surgical technique for the management of concealed penis in children producing predictable results and excellent satisfaction of the parents and patients.

Dr. Benzi and collegues from Brazil performed on page 749 (8) a interesting translational study about the Fowler-Stephens surgery and concluded that the upper portion of the abdominal testis in human fetuses had a higher concentration of vessels than the lower portion. These results suggest that manipulation of the lower end of the testis during Fowler-Stephens surgery should be avoided in order to preserve the collateral circulation.

Dr. Ditonno and Collegues from Italy permorfed on page 757 (9) a nice report about your experience in Single Port (SP) Robotic Pyeloplasty and concluded that the observed outcomes and potential benefits, combined with the adaptability of the SP platform, hold promising implications for the application of SP system in pyeloplasty treatment.

The Editor-in-chief expects everyone to enjoy reading.
